# Impairments of auditory scene analysis in posterior cortical atrophy

**DOI:** 10.1093/brain/awaa221

**Published:** 2020-09-01

**Authors:** Chris J D Hardy, Keir X X Yong, Johanna C Goll, Sebastian J Crutch, Jason D Warren

**Affiliations:** Dementia Research Centre, Department of Neurodegenerative Disease, UCL Queen Square Institute of Neurology, London, UK

**Keywords:** posterior cortical atrophy, auditory scene analysis, hearing, dementia, Alzheimer's disease

## Abstract

Although posterior cortical atrophy is often regarded as the canonical ‘visual dementia’, auditory symptoms may also be salient in this disorder. Patients often report particular difficulty hearing in busy environments; however, the core cognitive process—parsing of the auditory environment (‘auditory scene analysis’)—has been poorly characterized. In this cross-sectional study, we used customized perceptual tasks to assess two generic cognitive operations underpinning auditory scene analysis—sound source segregation and sound event grouping—in a cohort of 21 patients with posterior cortical atrophy, referenced to 15 healthy age-matched individuals and 21 patients with typical Alzheimer’s disease. After adjusting for peripheral hearing function and performance on control tasks assessing perceptual and executive response demands, patients with posterior cortical atrophy performed significantly worse on both auditory scene analysis tasks relative to healthy controls and patients with typical Alzheimer’s disease (all *P *<* *0.05). Our findings provide further evidence of central auditory dysfunction in posterior cortical atrophy, with implications for our pathophysiological understanding of Alzheimer syndromes as well as clinical diagnosis and management.

## Teaser

Posterior cortical atrophy (PCA) is thought of as a visual-led dementia. However, Hardy, Yong *et al.* show that people with PCA also have difficulties with auditory scene analysis – the central hearing processes that enable us to focus on a single person speaking in a crowded room – suggesting that PCA affects more than vision.

**See Särkäö and Sihvonen (doi:10.1093/brain/awaa218) for a scientific commentary on this article.**

## Introduction

Posterior cortical atrophy (PCA) is conventionally defined as a syndrome characterized by progressive impairment of higher visual function, in particular visuoperceptual and visuospatial skills, often designated the ‘visual variant’ of Alzheimer’s disease (Benson *et al.*, 1988; Crutch *et al.*, 2017). Patients with PCA have particular difficulty interpreting and navigating ‘busy’, dynamic visual environments requiring parsing of multiple objects distributed in space (Shakespeare *et al.*, 2013; Yong *et al.*, 2018). Posterior cortical functions such as calculation, spelling and praxis are also affected in PCA (Benson *et al.*, 1988; Crutch *et al.*, 2017), along with other cognitive domains including language (Crutch *et al.*, 2013), episodic memory (Ahmed *et al.*, 2018), working memory (Trotta *et al.*, 2019), executive functioning (Putcha *et al.*, 2018), and visuo-vestibular integration (Crutch *et al.*, 2018).

In the realm of sound, both PCA and typical amnestic Alzheimer’s disease adversely affect the processing of auditory spatial information, leading to impaired detection of sound source motion and localization of stationary sounds in space (Golden *et al.*, 2015). Such deficits are likely to contribute to impaired navigation of complex, everyday environments in both PCA and typical amnestic Alzheimer’s disease, and may form part of a wider spectrum of central auditory dysfunction in these syndromes (Crutch *et al.*, 2013; Slattery *et al.*, 2019). Taken together, this evidence suggests that central auditory impairment may be more significant in PCA than generally recognized.

The process of auditory scene analysis (ASA) depends fundamentally on accurate and efficient parsing of the auditory environment into its constituent sound sources and patterns (Bregman, 1994), such that these ‘auditory objects’ (Griffiths and Warren, 2004) can be tracked, analysed and ultimately associated with meaning. One striking example of ASA in action is the well-known ‘cocktail party effect’ (exemplified by hearing one’s own name across a crowded room); however, a broadly similar task confronts the brain whenever we process a target sound under the non-ideal listening conditions of daily life. Because everyday auditory environments usually contain multiple, acoustically diverse sound sources that are superimposed and evolving over time, ASA demands substantial neural computational resources: it is therefore potentially vulnerable to the early effects of neurodegenerative pathologies. Patients with typical amnestic Alzheimer’s disease have a generic deficit of ASA, linked to degeneration of posterior temporo-parietal networks (Goll *et al.*, 2012). On neuroanatomical and neuropathological grounds, impaired ASA is therefore anticipated to be a feature of PCA and indeed, might manifest earlier and more saliently in PCA than typical amnestic Alzheimer’s disease (Benson *et al.*, 1988; Crutch *et al.*, 2017; Firth *et al.*, 2019). However, it is challenging to assess perception of auditory scenes in the clinic or laboratory and ASA has not been investigated before in PCA.

Here we used a previously devised neuropsychological paradigm (Goll *et al.*, 2012) to assess ASA in patients with PCA, relative to patients with typical amnestic Alzheimer’s disease and healthy older individuals. This paradigm uses synthetic sound sources presented as dual auditory sequences to simulate the kinds of interactions between auditory objects that might occur in any natural auditory scene. Experimental tasks assessed two core, complementary cognitive operations underpinning ASA: ‘segregation’ (the parsing of superimposed, coincident sounds into separate sound objects, as in the cocktail party effect), and ‘grouping’ (the perceptual assembly of temporally-distributed sound events into a single auditory object, as when hearing out a melody for a particular voice or instrument in polyphonic music). We hypothesized that patients with PCA as a group would show significant deficits of ASA-segregation and ASA-grouping, relative both to healthy controls and to patients with typical amnestic Alzheimer’s disease.

## Materials and methods

Twenty-one patients with PCA, 21 patients with typical amnestic Alzheimer’s disease (published previously: Goll *et al.*, 2012) and 15 healthy age-matched individuals were included. All patients with PCA presented with visual difficulties and relative sparing of memory, language and insight, while patients with typical amnestic Alzheimer’s disease presented with episodic memory decline. Patients fulfilled criteria for the relevant diagnosis (Dubois *et al.*, 2007; Crutch *et al.*, 2017) (Supplementary Table 1), corroborated by general neuropsychological assessment, CSF examination and brain MRI. Participant group characteristics are summarized in Table 1; additional neuropsychological data for the PCA group are presented in Supplementary Table 2. Disease-related atrophy profiles were assessed using voxel-based morphometry for each patient group (details in the Supplementary material). Study approval was granted by the institutional ethics committee; all participants gave informed consent following Declaration of Helsinki guidelines.

**Table 1:** Summary of demographic, clinical, audiometric and auditory scene analysis task data for all participant groups

	Controls	PCA^a^	tAD
**General**			
*n*, male: female	7:8	7:14	9:12
Age, years	63.36 (6.4)	63.02 (9.5)	65.03 (7.9)
Hearing loss, dB	7.9 (7.7)	**17.0 (10.5)** *	13.6 (6.9)
Symptom duration, years	–	*3.65* (*2.3*)	5.93 (2.5)
**Background neuropsychology**			
MMSE (/30)	–	*18.48* (*4.6*)	22.10 (4.2)
RMT Words (*z*-score)^b^	–	**−2.15 (2.2)**	**−2.65 (1.9)**
RMT Faces (*z*-score)^c^	–	−1.95 (2.3)	**−2.28 (2.0)**
Digit span forward (/12)	8.40 (1.6)	**6.81 (1.9)**	7.48 (2.3)
Digit span reverse (/12)	7.27 (1.3)	***3.14* (*1.3*)**	**5.24 (2.8)**
WASI Vocabulary (/72)^d^	71.36 (4.4)	54.47 (8.8)**	**57.00 (14.8)**
Graded naming test (/30)^d^	27.00 (3.3)	**13.90 (4.6)**	**13.95 (9.0)**
Graded difficulty arithmetic (/24)^d^	15.55 (3.7)	***1.76* (*3.2*)**	**6.33 (4.9)**
Single word comprehension (*z*-score)	–	0.23 (0.7)	−6.41 (7.7)***
**ASA tests**			
ASA segregation^b^			
ASA segregation test (/20)	19.07 (1.5)	12.14 (3.0)****	**15.45 (4.2)**
Task requirement control test (/10)	10.00 (0.01)	***8.95* (*1.2*)**	10.00 (0.0)
Perceptual cue control test (/10)	9.67 (0.6)	8.71 (1.4)	9.35 (1.0)
ASA grouping			
ASA grouping test (/20)^c^	18.67 (1.2)	***11.05* (*3.8*)**	**15.62 (3.8)**
Task requirement control test (/10)	9.93 (0.3)	***9.00* (*0.8*)**	10.0 (0.0)
Perceptual cue control test (/10)	9.87 (0.4)	***7.90* (*1.9*)**	9.86 (0.5)

Mean (SD) data are presented unless otherwise indicated; maximum scores for neuropsychological tests are indicated in parentheses. Bold indicates significantly lower than healthy controls, *P *<* *0.001 unless otherwise specified (for *z*-scores, bold indicates average performance outside 95% of the area under normal distribution, i.e. ±1.96); italics indicate significantly lower than the typical Alzheimer’s disease (tAD) group, *P *≤* *0.01 unless otherwise specified (statistical data including 95% CIs are presented in full in Supplementary Table 3). Certain cognitive functions were assessed using different tests in the PCA and typical Alzheimer’s disease cohorts: the typical Alzheimer’s disease cohort was given the long-form Recognition Memory Test (RMT) for words and faces and the British Picture Vocabulary Scale (BPVS) for single word comprehension; the PCA group were given the short-form RMT for words and faces, and the Concrete Synonyms test for single word comprehension, and to give a comparable indication of how each syndromic group performed in these domains we derived *z*-scores using age-appropriate normative data (Supplementary Table 3). Administration of the graded naming test differed across groups: control and typical Alzheimer’s disease participants were presented with items visually; participants with PCA were asked to name from verbal description. MMSE = Mini-Mental State Examination score; WASI = Wechsler Abbreviated Scale of Intelligence.

^a^ CSF profiles of tau and amyloid-β were available for 13 patients with PCA and were consistent with Alzheimer’s pathology in 12 cases, based on local reference ranges (total tau/amyloid-β_1-42_ ratio > 1). One participant with PCA showed clear response bias on the ASA-grouping task (Supplementary material); this participant was removed from analysis of the ASA-grouping test.

^b^ Data were not available for one participant with typical Alzheimer’s disease.

^c^ Data were not available for one participant with PCA.

^d^ Data were not available for four healthy control participants.

* *P *=* *0.003 versus controls.

** *P *=* *0.002 versus controls.

*** *P *<* *0.001 versus PCA.

**** *P *=* *0.012 versus typical Alzheimer’s disease.

To assess any effects of hearing loss on performance in the experimental tasks, all subjects underwent pure tone audiometry, following a previously described procedure (Supplementary material). For each participant, the mean audiometric threshold over five frequencies (0.5, 1, 2, 3, 4 kHz) was taken as a composite score for that participant’s peripheral hearing function, used as a covariate in subsequent analyses.

**Figure 1:**
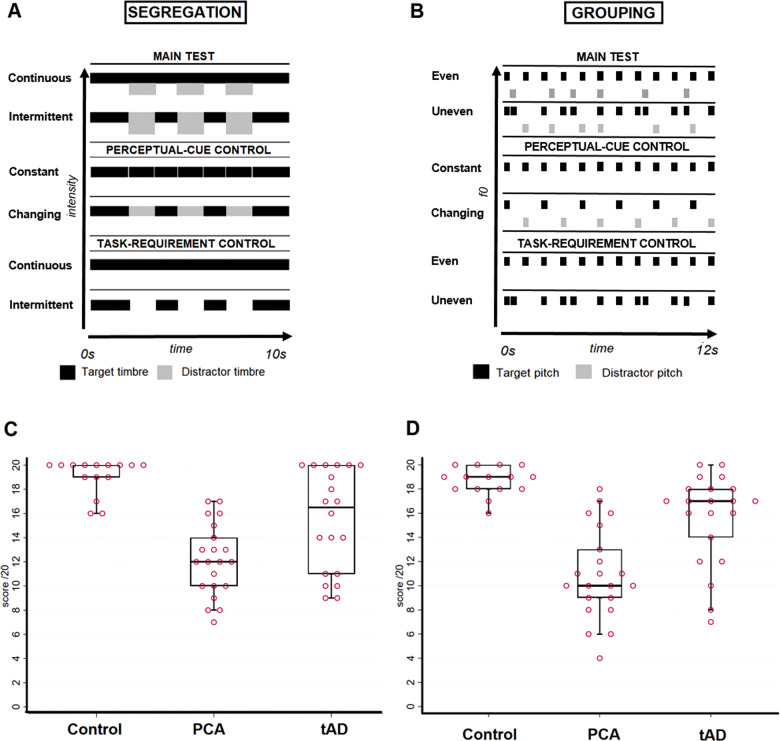
**Summary of the ASA paradigm and participant performance.** *Top* panels depict the ASA paradigms (**A** and **B**); *bottom* panels show plots of individual participant performance on the respective ASA tests (**C** and **D**); see also Table 1 and further stimulus details in the Supplementary material. Conditions in all subtests of the ASA-segregation assessment (**A**) and ASA-grouping assessment (**B**) are shown. Each *top* panel signifies one trial on which a sequence of sounds (harmonic complexes) was presented; rectangles represent individual sound elements (width indicates relative duration and height indicates relative intensity (**A**) or fundamental frequency (f0, corresponding to pitch; **B**). **A** and **B** are intended as illustrative diagrams, and not to scale; the same range of intensity and pitch values was used in each test. In the main ASA-segregation test (**A**), sound sequences (each of total duration 10 s) containing two different timbres (coded as black and light grey) were presented, and 20 trials comprising two experimental conditions were created by varying the temporal pattern such that one timbre (designated the ‘target’) was either continuous (10 trials) or intermittent (10 trials); the task on each trial was to decide whether the target sounds were ‘long’ (i.e. continuous) or ‘on-off’ (i.e. intermittent). A perceptual-cue control test was created to establish that participants were reliably able to detect timbre changes: 10 sound sequences were presented, five with continuous fixed timbre and five with two alternating timbres, and the task on each trial was to decide if the sound was ‘same’ or ‘changing’. A task-requirement control test was used to establish that participants could comply with the requirement to report continuous and intermittent temporal patterns: 10 sequences of sounds were presented, five continuous and five intermittent, and the task on each trial was to decide whether the sound was ‘long’ (i.e. continuous) or ‘on-off’ (i.e. intermittent). In the main ASA-grouping test (**B**), 20 trials (each of total duration 12 s) were presented, each comprising two interleaved sound sequences with two different pitch values (coded as black and light grey; one designated the ‘target’ pitch) such that the target pitch sequence was either isochronous (fixed inter-sound interval, 10 trials) or anisochronous (randomly varying inter-sound interval, 10 trials); the task on each trial was to decide whether sounds with the target pitch were ‘even’ or ‘uneven’. A perceptual-cue control test was created to establish that participants were reliably able to detect pitch differences: 10 isochronous sequences were presented, five with fixed pitch and five with changing pitch, and the task on each trial was to decide if the pitch was ‘same’ or ‘changing’. A task-requirement control test was used to establish that participants could comply with the requirement to report even and uneven temporal patterns: 10 sequences of sounds with fixed pitch were presented, five isochronous and five anisochronous, and the task on each trial was to decide whether the sequence was ‘even’ or ‘uneven’. Participants were familiarized with the task and the targets for each test beforehand, to ensure they fully understood the experimental instructions (Supplementary material). Data plots show individual scores (circles) on the ASA-segregation main task (**C**) and ASA-grouping main task (**D**). For each group, horizontal lines indicate median score, oblongs code interquartile range and whiskers 95% confidence intervals; a score of 10 would correspond to chance performance. Control = healthy control group; PCA = patient group with posterior cortical atrophy; tAD = patient group with typical Alzheimer’s disease.

Auditory scene analysis was assessed using two main experimental tasks, as described previously (Goll *et al.*, 2012) (details of tasks and stimuli are provided in the Supplementary material and Supplementary Fig. 1): an ASA-segregation task, requiring separation of temporally concurrent sound objects based on spectral shape (an important acoustic cue to the perception of timbre, or sound identity; Fig. 1A); and an ASA-grouping task, requiring grouping of temporally spaced sound objects into a single stream on the basis of fundamental frequency (an important acoustic determinant of the perception of pitch; Fig. 1B). Prior to each of these ASA tests, we administered bespoke control tests using tasks designed to familiarize participants with the paradigm and to assess more general cognitive processes that might affect ASA: a ‘perceptual-cue’ control, to establish how well participants could discriminate changes in perceptual cues (timbre or pitch) driving the relevant ASA task; and a ‘task-response’ control, to establish how well participants could comply with the general response and working memory requirements of the relevant ASA test. All participants completed initial practice trials and only commenced the experimental tests once the experimenter was confident that participants understood the relevant tasks (Supplementary material).

The task on each trial was a two-alternative forced-choice decision. For the segregation task, participants were asked to report verbally whether target stimuli were ‘long’ (i.e. continuous) or ‘on-and-off’ (intermittent); for the grouping task, participants reported whether target stimuli were even or uneven (Fig. 1A and B). Details of the experimental protocol and examples of the stimuli are provided in the Supplementary material; all sounds were presented from a notebook computer binaurally via headphones at a comfortable listening level. No feedback about performance was given during the assessment and no time limit was imposed on subject responses.

Data were analysed in Stata version 14 (StataCorp). Demographic and clinical characteristics were compared between groups using ANOVA and Fisher’s exact tests; where the omnibus test was significant, *post hoc* comparisons between groups were investigated. Scores on each of the ASA and control tests were entered into separate analysis of covariance (ANCOVA) regression models, adjusting for peripheral hearing score and (for the ASA tests) also for corresponding control task scores; importantly, these control tasks were similar to the ASA tasks on more general perceptual, attentional, working memory, recall and response decision demands, allowing us to take account of these potentially confounding factors in the ASA group comparison. Pairwise group differences were assessed using planned comparisons that also adjusted for these covariates. Residuals from the ANCOVA models were not normally distributed, so we adopted a non-parametric approach that allowed for relaxation of the normality and heteroscedasticity assumptions made by ANCOVA; full details of this approach are given in the Supplementary material. We used Spearman’s ρ to assess associations between performance on each of the main ASA tasks and Mini-Mental State Examination (MMSE) in the PCA group. An alpha level of 0.05 was accepted as the statistical significance threshold for all tests.

### Data availability

The data that support the findings of this study are available on request from the corresponding author. The data are not publicly available as they include information that could compromise the privacy of the research participants.

## Results

Results for group comparisons of demographic and clinical characteristics and experimental test performance are summarized in Table 1 and additional data are presented in Supplementary Tables 2 and 3; individual participant performance plots on the ASA tests are shown for each participant group in Fig. 1C and D. Syndromic groups showed the anticipated profiles of disease-related brain atrophy (Supplementary Fig. 1).

Participant groups did not differ significantly in age [*F*(2,54) = 0.35, *P = *0.71] or gender (*P = *0.79). There was evidence of an overall difference in peripheral hearing function across groups [*F*(2,54) = 4.92, *P = *0.01], with the PCA group performing more poorly than healthy controls (*t* = −3.13, *P = *0.003). Relative to the typical amnestic Alzheimer’s disease group, the PCA group had a significantly shorter symptom duration [*t*(40) = −3.09, *P = *0.004] despite a lower MMSE score [*t*(40) = −2.66, *P = *0.01]. The PCA group performed significantly more poorly than both the healthy control group and the typical amnestic Alzheimer’s disease group on the bespoke tests controlling for task response and working memory demands and on one test controlling for perceptual discrimination ability (details in Table 1 and Supplementary Table 1).

After adjusting for group differences in peripheral hearing function and control task performance, there was a significant group effect on performance in the main ASA-segregation test [*F*(2,50) = 12.12, *P < *0.001], the PCA group performing worse than both the healthy control group {adjusted difference, −6.40 [95% confidence intervals (CI): −8.54 to −4.41], *t* = −4.90, *P < *0.001} and the typical amnestic Alzheimer’s disease group [adjusted difference, −3.10 (95% CI: −5.49 to −0.74), *t* = −2.63; *P *=* *0.01].

There was also an overall group effect on performance in the main ASA-grouping test [*F*(2,50) = 8.91, *P *=* *0.001], the PCA group again performing worse than both the healthy control group [adjusted difference, −6.91 (95% CI: −11.27 to −3.48), *t* = −4.17, *P < *0.001] and the typical amnestic Alzheimer’s disease group [adjusted difference, −3.97 (95% CI: −8.12 to −0.14], *t* = −2.59; *P *=* *0.01]. Within the PCA group, performance on the main ASA tasks was not significantly associated with MMSE score (ASA-segregation, ρ  =  0.31, *P *=* *0.17; ASA-grouping, ρ  =  0.25, *P *=* *0.28). An analysis of hit rates for different sequence types in the main ASA tests within each patient group did not find evidence of response bias (Supplementary Table 4).

## Discussion

We have presented evidence that patients with PCA perform worse on tasks of ASA-segregation and ASA-grouping than healthy older individuals or patients with typical amnestic Alzheimer’s disease. Our data suggest that this impairment is not attributable to peripheral hearing function, executive task demands or working memory capacity, nor to general disease severity. Rather, the findings support the concept of a multi-sensory breakdown in parsing the world at large in PCA.

Our findings corroborate earlier work suggesting that there may be a prominent auditory contribution to the PCA phenotype (Crutch *et al.*, 2013; Golden *et al.*, 2015; Slattery *et al.*, 2019). The ASA-segregation and ASA-grouping tasks used here were developed to probe fundamental processes in the disambiguation and understanding of auditory scenes. We have previously shown that patients with typical amnestic Alzheimer’s disease are impaired on these tasks relative to healthy individuals of similar age (Goll *et al.*, 2012). Current findings emphasize that these generic ASA component processes are substantially more severely impaired in PCA, underlining the profound involvement of posterior cortical functions in this syndrome. ASA impairment may have a common neuroanatomical and pathophysiological basis in PCA and typical amnestic Alzheimer’s disease, linked to involvement of the core temporo-parietal ‘default mode’ network (Greicius *et al.*, 2004; Goll *et al.*, 2012; Crutch *et al.*, 2017); ASA may engage a generic interface between external sensory events and the internal monitoring and modelling of those events over time, instantiated by this network (Goll *et al.*, 2012).

In addition to more severe ASA impairment, the PCA cohort here exhibited deficits of more elementary auditory processing not clearly apparent in patients with typical amnestic Alzheimer’s disease: namely, pure tone detection and pitch change perception. To our knowledge, audiometric deficits have not previously been identified as a feature of PCA but this corroborates emerging evidence that pure tone detection can be impaired even in canonical dementia syndromes (most notably, non-fluent/agrammatic variant primary progressive aphasia) that typically spare the peripheral auditory pathways (Hardy *et al.*, 2016, 2019). The processing of pitch change (particularly in the context of a task requiring working memory) is likely to depend on temporo-parietal junctional cortices that are targeted in PCA (Crutch *et al.*, 2012; Plack *et al.*, 2014; Golden *et al.*, 2016; Firth *et al.*, 2019; Phillips *et al.*, 2019): impaired pitch processing here may also indicate a more general impairment of magnitude estimation, as patients with PCA also have difficulties with other aspects of numerical and spatial magnitude encoding (Delazer *et al.*, 2006; Spotorno *et al.*, 2014; González *et al.*, 2019) and these processes are likely to depend on neural mechanisms that are at least partly shared across modalities (Kadosh *et al.*, 2008; Bonn and Cantlon, 2012).

PCA participants were also impaired relative to healthy controls and typical amnestic Alzheimer’s disease participants on both control tasks controlling for specific task demands. Performance on these tasks was incorporated in the main analyses of ASA-segregation and ASA-grouping, but this deficit remains noteworthy. One parsimonious explanation may be that these tasks tax auditory working memory processes more affected in the PCA group (as indexed by reverse digit span, Table 1).

From a clinical standpoint, these findings support the bedside impression that people with PCA have difficulty following speech and other sounds of interest particularly in busy auditory environments, and suggest that defective ASA is an even more salient limitation in PCA than in typical amnestic Alzheimer’s disease. However, further work is required to characterize the auditory phenotype of PCA more fully, and to evaluate the extent to which ASA impairments constitute a hallmark of Alzheimer’s disease pathology; CSF data were only available for 13 patients (62%), meaning that we lacked confirmation of pathophysiology in the remaining eight cases. Emerging evidence suggests that the logopenic variant of primary progressive aphasia, which is typically associated with Alzheimer’s disease pathology (Spinelli *et al.*, 2017), may have a specific auditory phenotype (Hardy *et al.*, 2018; Johnson *et al*., 2020); it remains to be seen whether ASA might also be affected in this patient population. Deficits detected on these psychoacoustic tests across the Alzheimer’s disease spectrum should be correlated with structural and functional neuroimaging data to define their neural mechanisms.

In larger patient cohorts and future longitudinal studies, it will be of interest to determine how early auditory scene analysis deficits emerge, with a view to advancing diagnosis; and whether different subsyndromes of PCA [including those attributable to non-Alzheimer pathologies (Crutch *et al.*, 2017)] have distinct auditory phenotypes, with neuropathological correlation. Finally, adopting a therapeutic perspective, there is the promise [based on work in typical amnestic Alzheimer’s disease (Hardy *et al.*, 2017, 2018)] that residual auditory plasticity might be harnessed in PCA to benefit hearing under challenging listening conditions, via cognitive training and pharmacological modulation with acetylcholinesterase inhibitors. The deficits observed here should be compared with daily life symptoms of communication and disease burden to assess their functional relevance more directly. Strategies to support everyday activities (such as reading) in PCA often rely on auditory presentation. Understanding how to optimally combine auditory, visual, and other sensory cues may offer key insights in enabling navigation of the world at large by people with PCA and other forms of dementia.

## Supplementary Material

awaa221_Supplementary_Data

## Abbreviations

ASA =: auditory scene analysis;
PCA =: posterior cortical atrophy
