# Supplementary material for: Impairments of auditory scene analysis in posterior cortical atrophy
Source: Brain. 2020 Sep 1;143(9):2689–95. doi: 10.1093/brain/awaa221 (PMC7523698; doi:10.1093/brain/awaa221)
Supplement: awaa221_Supplementary_Data [file awaa221_supplementary_data.zip › awaa221-suppl-data/brain-2020-00474-File004.pdf]

SUPPLEMENTARY MATERIAL. Impairments of auditory scene analysis in posterior cortical atrophy, by CJD Hardy et al.

Assessment of peripheral hearing

To assess any effects of hearing loss on performance in the experimental tasks, all participants underwent pure tone audiometry, administered via headphones from a notebook computer in a quiet room. The procedure was adapted from a commercial screening audiometry software package (AUDIO-CDTM, Digital Recordings, http://www.digital-recordings.com/audiocd/audio.html). Five frequencies (0.5, 1, 2, 3 and 4 kHz) were assessed: at each frequency, subjects were presented with a continuous tone that slowly and linearly increased in level in dB (note that this adapted audiometric protocol was not intended to be equivalent to standard, clinical audiometry). Subjects were instructed to tap as soon as they could detect the tone; this response time corresponds to the level (dB) of the tone at the point at which it is heard. The mean value for three presentations of the same tone in the right ear (or the left ear in the case of one patient with Alzheimer's disease who reported unilateral right-sided hearing loss) was taken as the detection threshold for that frequency.

Assessment of auditory scene analysis: stimuli and experimental conditions

Experimental stimuli, tasks and controls are diagrammed schematically in Figure 1. All auditory stimuli were created digitally in Matlab 7.2® (The MathWorks, Inc.).

Acoustic parameters of stimulus sound elements. The experimental stimuli used in this study comprised sequences of sounds: in all tests, the individual elements of these sequences were synthetic sound ‘objects’ (schematised in Figure S1) created by manipulating the harmonic series based on a fundamental frequency (f0) with specified frequency envelope (‘spectral shape’, corresponding to the relative intensities of the component harmonics) and temporal envelope (temporal ‘shape’, corresponding to the structure of the sound in time). Across the experimental tests here, sound elements had a frequency spectrum bounded by lower cut-off frequency f0 and upper cut-off frequency 3000 Hz. In the temporal domain, sound elements were all linearly windowed on and off over 30 ms and amplitude modulated (rate 80 Hz, depth 30%); the characteristic of amplitude modulation was task-irrelevant here and purely empirical, as we have found that it enhances the subjective pleasantness and salience of synthetic sound stimulus identity for participants.

To create different sound elements for each of the experimental conditions in the ASA tests (described below), we varied f0 or spectral envelope (by variably attenuating blocks of harmonics within the series by 0 – 20 dB; this effectively changed the interpolated spectral ‘shape’ of the harmonic series, see Figure S1). The physical acoustic property of f0 is a major determinant of the percept of pitch: that attribute of auditory sensation in terms of which sounds may be ordered on a frequency-related (e.g., musical) scale. The acoustic characteristic of spectral shape is a major determinant of timbre: that attribute which distinguishes two different sounds of identical pitch, duration and intensity (e.g., the same note played on a flute and a clarinet). Timbre is therefore an important cue to the identity of a sound; and both pitch and timbre are integral to parsing the sound objects and information streams that compose auditory scenes[1].

In this experiment, by varying the acoustic characteristics, f0 and spectral shape of the stimulus sound elements, we were correspondingly varying their perceived pitch and timbre, respectively; however, f0 should not be equated to pitch nor spectral shape to timbre. Both pitch and timbre are complex percepts that are influenced by various acoustic cues: for example, pitch can be conveyed by periodic (temporal) repetition as well as by frequency (spectral) information, while timbre is a multidimensional property determined by both spectral and temporal information as well as their dynamic interaction or ‘flux.’

ASA-segregation tests (sound stimulus examples 1–6). For the main ASA-segregation test, stimuli (20 trials) were created by superimposing two sequences of sound elements to create composite continuous sounds each with overall duration 10 s. On every trial, one sound sequence had a timbre designated as the 'target' timbre, Tt, while the other had a distinct 'distractor' timbre, Td. Four different distractor timbres, each distinct from Tt, were randomly distributed across the stimulus set to guard against any idiosyncratic effects that might follow the superposition of a particular timbre pair. On every trial, the Td sequence comprised 1 s intervals of sound separated by 1 s inter-sound gaps. Two experimental conditions were created by varying the temporal pattern of the Tt sequence, which was either continuous over the whole trial (10 trials) or intermittent with 1 s intervals of sound separated by 1 s inter-sound gaps mirroring the temporal pattern of the Td sequence (10 trials). In the 'intermittent' condition, the intensity level of Td was increased to match the overall intensity level in the 'continuous' condition. The task on each trial was to decide whether Tt sounds were 'long' (i.e. continuous) or 'on-off' (i.e. intermittent).

A perceptual-cue control test was created to establish that participants were reliably able to detect timbre changes. Ten sound sequences were presented, five with continuous fixed timbre Tt, and five with timbre alternating between Tt and Td. The four distinct Td timbres described above were randomly distributed across the latter five stimuli; across the set of sequences, the temporal patterns used matched those of the sequences in the main test. The task on each trial was to decide if the sound was 'same' or 'changing'.

In addition, a task-requirement control test was used to establish that participants could comply with the requirement to report continuous and intermittent temporal patterns. Ten sequences of sounds with timbre Tt were presented, five continuous and five intermittent; the temporal pattern of sequences matched those used in the main test. The task on each trial was to decide whether the sound was 'long' (i.e. continuous) or 'on-off' (i.e. intermittent).

ASA-grouping tests (sound stimulus examples 7–12). For the main ASA-grouping test, stimuli (20 trials) were created by interleaving two sequences of sound elements to create composite sound sequences, each with overall duration 12 s; each individual sound element in a sequence had duration 60 ms. Individual sounds were assigned one of two pitches, either a 'target' f0 (Pt = 423 Hz) or a 'distractor' f0 (Pd = 237 Hz); these f0 values were chosen such that they did not align with any familiar tonal interval from western musical scales. To create two experimental conditions, the presentation of sound elements with f0 Pt was either isochronous (fixed inter-sound interval 135 ms; the 'even' condition) or anisochronous (inter-sound interval varying pseudo-randomly between 210 and 930 ms; the 'uneven' condition). The overall temporal distribution of sound elements (both Pt and Pd) and the mean rate of presentation of sounds with f0 Pt were matched between conditions. Each condition comprised 10 trials; the task on each trial was to decide whether Pt sounds were 'even' (i.e. isochronous) or 'uneven' (i.e., anisochronous).

A perceptual-cue control test was created to establish that subjects were reliably able to detect f0 differences. Ten isochronous sequences were presented, five with f0 fixed at Pt and five with f0 changing between Pt and Pd; the tempi of the sequences matched those used in the main test. The task on each trial was to decide if the f0 was 'same' or 'changing'.

In addition, a task-requirement control test was used to establish that subjects could comply with the requirement to report even and uneven temporal patterns. Ten sequences of sounds with f0 Pt were presented, five isochronous and five anischronous; the temporal pattern of sequences matched those used in the main test. The task on each trial was to decide whether the sequence was 'even' (i.e. isochronous) or 'uneven' (i.e. anisochronous).

Assessment of auditory scene analysis: practice phase and experimental protocol

Participants were familiarised with task requirements prior to each test (using example stimuli not administered during the subsequent assessment). There were two practice trials per control task, and four per main task. If the participant made a mistake on a practice trial, feedback was given,

and practice trials were repeated if necessary. Testing was only commenced once the experimenter was confident that the participant understood what was being asked of them. The testing sessions were short with breaks between the component tasks to reduce any potential for fatigue or forgetting task instructions.

During the main experiment, all sounds were presented as digital wavefiles from a notebook computer binaurally via Sennheiser HD 280-Pro headphones, at a comfortable sound pressure level in the range 70 - 75 dB, as measured using a hand-held sound level meter; the sound level was initially set according to individual preference but held constant for a particular participant throughout the experiment. Each ASA assessment was administered in a fixed order: perceptual-cue control, task-requirement control, main test. Within each subtest, trials were presented in a fixed randomised order. Verbal responses were recorded for off-line analysis. No feedback about performance was given during the assessment and no time limit was imposed on participant responses.

Brain image acquisition and analysis: disease-related atrophy profiles

T1-weighted volumetric magnetic resonance images were acquired on a Siemens Trio TIM 3T scanner (Siemens Medical Systems) for 20 patients with PCA. Scans for the 20 participants with tAD had been collected and reported previously[2]. Images for all participants were acquired using a 3D magnetization prepared rapid gradient echo (MP-RAGE) sequence producing 208 contiguous 1.1 mm thick sagittal slices with 28-cm field of view and a 256 × 256 acquisition matrix, giving approximately isotropic 1.1 mm cubic voxels; a 32-channel head coil was used (apart from two subjects in the tAD group for whom a 12-channel coil was used).

We used voxel-based morphometry (VBM) to generate disease-associated atrophy profiles for the two patient groups. Brain images were pre-processed and normalised to Montreal Neurological Institute space with isotropic voxel size 1.5mm using SPM12 software http://www.fil.ion.ucl.ac.uk/spm/software/spm12/) and the Diffeomorphic Anatomical Registration Through Exponentiated Lie Algebra (DARTEL) toolbox with default parameters in MATLAB R2014b (MathWorks, Natick, MA, USA), using a Gaussian smoothing kernel of 6mm full-width at half-maximum in all directions. For visualisation of results, a disease-specific mean structural brain image template was created by warping all bias-corrected native space whole brain images to the final DARTEL template and calculating the average of the warped images. An explicit mask was created for each patient group using an automatic mask creation strategy so that only appropriate voxels would be included in resultant analyses[3].

We then used a multiple regression model incorporating covariates of age, gender, and total intracranial volume (TIV), exploring voxel intensity (an index of grey matter volume) as a function of diagnosis, to contrast the two syndromic groups and identify regions of differential atrophy in PCA and tAD. TIV was calculated by adding together grey matter, white matter, and CSF volumes generated during the initial segmentation step[4]. T-contrast whole-brain unthresholded effect maps were generated by using the ‘slover’ command in SPM12, and overlayed on the mean structural template. They are presented in Figure S2.

Non-parametric analysis of behavioural data

We adopted a permutation approach attributable to Freedman & Lane[5]. This approach gives the same parameter estimates as ANCOVA but with p-values relaxing the normality and homoscedasticity assumptions made by ANCOVA; the p-values reported here were computed using 50000 permutations of residuals from models adjusting for hearing (control tasks) and models adjusting for hearing and associated control task scores (main tests). We also calculated non-parametric bias-corrected and accelerated-bootstrap confidence intervals[6] for the between group differences based on 50000 bootstrap resamples; these also relax assumptions of normality and homoscedasticity.

Assessment of patient response bias

To assess whether performance scores were balanced for even versus uneven responses in the ASA-grouping task and continuous vs intermittent responses in the ASA-segregation task, we compared ‘hit rates’ within each participant group for each sequence type. For a given sequence type, we took the number of times each participant had correctly identified this sequence, e.g. “even” as “even”, and divided this by 10 (the actual number of each stimulus type) to create hit rates. We then used paired two-tailed t-tests to assess statistically whether, within each participant group, participants showed a performance advantage for one type of sequence. Results from these analyses are given in Table S3.

References

1. Bregman (1994) Auditory Scene Analyis: Perception of organization of sound. MIT Press, Cambridge, Massachusetts
2. Goll JC, Kim LG, Ridgway GR, et al (2012) Impairments of auditory scene analysis in Alzheimer’s disease. Brain 135:190–200. doi: 10.1093/brain/awr260
3. Ridgway GR, Omar R, Ourselin S, et al (2009) Issues with threshold masking in voxel-based morphometry of atrophied brains. Neuroimage 44:99–111. doi: 10.1016/j.neuroimage.2008.08.045
4. Malone IB, Leung KK, Clegg S, et al (2015) Accurate automatic estimation of total intracranial volume: a nuisance variable with less nuisance. Neuroimage 104:366–72. doi: 10.1016/j.neuroimage.2014.09.034
5. Freedman D, Lane D (1983) A Nonstochastic Interpretation of Reported Significance Levels. J Bus Econ Stat 1:292–298. doi: 10.1080/07350015.1983.10509354
6. Efron B, Tibshirani R (1994) An introduction to the bootstrap. Chapman & Hall/CRC, Boca Raton, Florida
7. Mendez MF, Ghajarania M, Perryman KM (2002) Posterior Cortical Atrophy: Clinical Characteristics and Differences Compared to Alzheimer’s Disease. Dement Geriatr Cogn Disord 14:33–40. doi: 10.1159/000058331
8. Tang-Wai DF, Graff-Radford NR, Boeve BF, et al (2004) Clinical, genetic, and neuropathologic characteristics of posterior cortical atrophy. Neurology 63:1168–1174. doi: 10.1212/01.WNL.0000140289.18472.15
9. Baxter D, Warrington E (1994) Measuring dysgraphia: a graded difficulty spelling test. Behav Neurol 7:107–16.
10. Warrington EK, James M (1991) The Visual Object and Space Perception Battery. Thames Valley Test Company, Bury St Edmunds, UK
11. Willison JR, Warrington EK (1992) Cognitive retardation in a patient with preservation of psychomotor speed. Behav Neurol 5:113–116. doi: 10.3233/BEN-1992-5207
12. James M, Plant GT (Gordon T, Warrington EK, Thames Valley Test Company. (2001) Corvist : cortical vision screening test : manual and test materials. Thames Valley Test Co, Bury St. Edmunds
13. Folstein M, Folstein S, McHugh P (1975) “Mini-mental state”: a practical method for grading the cognitive state of patients for the clinician. J Psychiatr Res 12:189–198.
14. Warrington EK (1996) The Camden Memory Test Battery. Psychology Press, Hove, UK.

Table S1. Diagnostic criteria followed for the posterior cortical atrophy group

**Mendez et al (2002)**[7]	**Tang-Wai et al. (2004)**[8]	**Crutch et al. (2017) Classification Level 1**
**Core diagnostic features (all must be present)**		**Clinical features**
A. Insidious onset, gradual progression<br>B. Presentation with visual complaints with intact primary visual functions<br>C. Evidence of predominant complex visual disorder on examination:<br>• Elements of Balint's syndrome<br>• Visual agnosia<br>• Dressing apraxia<br>• Environmental disorientation<br>D. Proportionally less impaired deficits in memory and verbal fluency<br>E. Relatively preserved insight with or without depression	A. Insidious onset, gradual progression<br>B. Presentation of visual complaints in absence of significant primary ocular disease explaining the symptoms<br>C. Relative preservation of anterograde memory and insight early in disorder<br>D. Disabling visual impairment throughout disorder<br>E. Absence of stroke or tumor<br>F. Absence of early parkinsonism and hallucinations<br>G. Any of the following findings:<br>• Simultanagnosia with/without optic ataxia or ocular apraxia<br>• Constructional dyspraxia<br>• Visual field defect<br>• Environmental disorientation<br>• Any element of Gerstmann syndrome	A. Insidious onset<br>B. Gradual progression<br>C. Prominent early disturbance of visual ± other posterior functions<br>**Cognitive features**<br>At least three of the following must be present as early or presenting features ± evidence of their impact on activities of daily living:<br>A. Space perception deficit<br>B. Simultanagnosia<br>C. Object perception deficit<br>D. Constructional dyspraxia<br>E. Environmental agnosia<br>F. Oculomotor apraxia<br>G. Dressing apraxia<br>H. Optic ataxia<br>I. Alexia<br>J. Left/right disorientation<br>K. Acalculia<br>L. Limb apraxia (not limb-kinetic)<br>M. Apperceptive prosopagnosia<br>N. Agraphia<br>O. Homonymous visual field defect<br>P. Finger agnosia<br>All of the following must be evident:<br>A. Relatively spared anterograde memory function<br>B. Relatively spared speech and nonvisual language functions<br>C. Relatively spared executive functions<br>D. Relatively spared behavior and personality
**Supportive diagnostic features**	**Supportive diagnostic features**	**Neuroimaging**
A. Presenile onset<br>B. Alexia<br>C. Elements of Gerstmann's syndrome<br>D. Ideomotor apraxia<br>E. Physical exam within normal limits<br>F. Investigations<br>• Neuropsychology: predominantly impaired perceptual deficits<br>• Brain imaging: predominantly occipitoparietal abnormality (especially functional neuroimaging) with relative sparing of frontal and mesiotemporal regions	A. Alexia<br>B. Presenile onset<br>C. Ideomotor or dressing apraxia<br>D. Prosopagnosia	Predominant occipito-parietal or occipito-temporal atrophy/ hypometabolism/ hypoperfusion on MRI/ FDG-PET/ SPECT
	**Investigations**	**Exclusion criteria**
	A. Neuropsychological deficits referable to parietal and/or occipital regions<br>B. Focal or asymmetric atrophy in parietal and/or occipital regions on structural imaging<br>C. Focal or asymmetric hypoperfusion/ hypometabolism in parietal and/or occipital regions on functional imaging	A. Evidence of brain tumor or other lesion sufficient to explain symptoms<br>B. Evidence of significant vascular disease including focal stroke sufficient to explain the symptoms<br>C. Evidence of afferent visual cause (optic nerve, chiasm, tract)<br>D. Evidence of other causes for cognitive impairment

The Table summarises the criteria used to assess patients with posterior cortical atrophy in this study. All patients satisfied Mendez criteria for PCA.Twenty patients also satisfied Tang Wai criteria: one patient did not on the basis that they had features of Parkinsonism. All patients satisfied Classification Level 1 of the consensus criteria published in 2017.

Table S2. Full background neuropsychological assessment scores for the posterior cortical atrophy group

	N	Mean	SD	Number of patients performing ≤ 5th %ile
**General psychology**				
MMSE	21	18.48	4.58	-
sRMT-words	21	19.71	3.93	7
Concrete synonyms	21	21.48	2.20	1
**Non-visual parietal**				
GDST[9] (/20)	21	9.19	6.30	4
Gesture production (/15)[a]	21	12.00	3.79	13
**Early visual**				
Crowding (/10)[a]	19	9.26	1.33	5
Figure-ground[10] (VOSP) (/20)	21	16.33	2.54	15
**Visuospatial**				
Number location (VOSP)[10] (/10)	19	2.16	2.75	17
Dot counting (VOSP)[10] (/10)	21	3.71	3.07	18
A cancellation (max 90s)[11]	20	79.8	22.8	19
**Visuoperceptual**				
Object decision[10] (VOSP) (/20)	19	11.16	4.73	19
Fragmented letters[10] (VOSP /20)	18	6.00	6.42	16
Unusual views (/20)	19	5.11	5.10	17
Usual views (/20)	19	12.05	7.12	17

The table shows the performance of the PCA cohort on a range of tests demonstrating impairment in key domains associated with PCA. The tAD cohort who completed the auditory scene analysis tasks did not complete the same battery of the neuropsychological tests. Maximum scores are shown in parentheses; the right-most column gives the number of participants in the cohort who scored at or below the 5th percentile based on published normative data. CORVIST, Cortical Visual Screening Test[12]; GDST, Graded Difficulty Spelling Test[9] (Part B); MMSE, Mini-Mental State Examination[13]; NA, not administered; sRMT, short Recognition Memory Test[14]; VOSP, Visual Object and Space Perception Battery[10]. [a]Test unpublished so normative data not available, but healthy control participants score at ceiling

Table S3. Demographic, clinical, audiometric, and auditory scene analysis task data and summary statistics for all participant groups, with group comparisons

	Controls	PCA*	tAD	PCA vs controls	tAD vs controls	PCA vs tAD	p-value
**General**							
N (M:F)	7:8	7:14	9:12	-	-	-	0.79
Age (yrs)	63.36 (6.4)	63.02 (9.5)	65.03 (7.9)	-	-	-	0.71
Hearing loss (dB)	7.9 (7.7)	**17.0 (10.5)[a]**	13.6 (6.9)	t=3.13, p=0.003	t=1.94, p=0.06	t=1.31, p=0.20	0.01
Symptom duration (years)	-	*3.65 (2.3)*	5.93 (2.5)	N/A	N/A	t=-3.09, p=0.004	0.004
**Background neuropsychology**							
MMSE (/30)	-	*18.48 (4.6)*	22.10 (4.2)	N/A	N/A	t=-2.66, p=0.01	0.01
RMT Words (z-score)[1]	-	**-2.15 (2.2)**	**-2.65 (1.9)**	-	-	t=0.78, p =0.44	0.44
RMT Faces (z-score)[2]	-	*-1.95 (2.3)*	**-2.28 (2.0)**	-	-	t=0.48, p=0.64	0.64
Digit span forwards (/12)	8.40 (1.6)	**6.81 (1.9)**	7.48 (2.3)	-	-	-	0.065
Digit span backwards (/12)	7.27 (1.3)	***3.14 (1.3)***	**5.24 (2.8)**	t=-6.14, p<0.001	t=-3.02, p=0.004	t=-3.42, p<0.001	<0.001
WASI Vocabulary (/72)[3]	71.36 (4.4)	**54.47 (8.8)**	**57.00 (14.8)**	t=-3.20, p=0.002	t=-3.44, p=0.001	t=0.13, p=0.89	0.003
Graded naming test (/30)[3]	27.00 (3.3)	**13.90 (4.6)**	**13.95 (9.0)**	t=-5.37, p<0.001	t=-5.36, p<0.001	t=0.02, p=0.98	<0.001
Graded difficulty arithmetic (/24)[3]	15.55 (3.7)	***1.76 (3.2)***	**6.33 (4.9)**	t=-9.22, p<0.001	t=-3.69, p=0.001	t=-6.16, p<0.001	<0.001
Single word comprehension (z-score)	-	0.23 (0.73)	-6.41 (7.66)	-	-	t=3.95, p < 0.001	<0.001
**ASA tests**							
ASA-segregation[1]							
ASA-segregation test (/20)	19.07 (1.5)	***12.14 (3.0)***	**15.45 (4.2)**	t=-4.90, p<0.001 -6.40 (-8.54 to -4.41)	t=-3.48, p<0.001 -3.29 (-5.40 to -1.54)	t=-2.63, p=0.012 -3.10 (-5.49 to -0.74)	<0.001
Task requirement control test (/10)	10.00 (0.00)	***8.95 (1.2)***	10.00 (0.0)	t=-3.51, p=0.001 -0.92 (-1.40 to -0.56)	t=0.28, p=0.78 0.07 (-0.01 to 0.37)	t=-4.36, p<0.001 -0.99 (-1.54 to -0.63)	<0.001
Perceptual cue control test (/10)	9.67 (0.6)	8.71 (1.4)	9.35 (1.0)	-	-	-	0.17
ASA-grouping							
ASA-grouping test (/20)[2]	18.67 (1.2)	***11.05 (3.8)***	**15.62 (3.8)**	t=-4.17, p<0.001 -6.91 (-11.27 to -3.48)	t=-2.98, p=0.001 -2.94 (-4.95 to -1.39)	t=-2.59, p=0.01 -3.97 (-8.12 to -0.14)	0.001
Task requirement control test (/10)	9.93 (0.3)	***9.00 (0.8)***	10.0 (0.0)	t=-5.02, p<0.001 -0.98 (-1.37 to -0.51)	t=0.22, p=0.83 0.04 (-0.14 to 0.31)	t=-6.13, p<0.001 -1.02 (-1.38 to -0.68)	<0.001
Perceptual cue control test (/10)	9.87 (0.4)	***7.90 (1.9)***	9.86 (0.5)	t=-4.88, p<0.001 -2.15 (-3.39 to -1.26)	t=-0.29, p=0.77 -0.13 (-0.59 to 0.24)	t=-5.38, p<0.001 -2.02 (-3.04 to -1.25)	<0.001

Certain cognitive functions were assessed using different tests in the PCA and tAD cohorts: the tAD cohort was given the long-form Recognition Memory Test (RMT) for words and faces and the British Picture Vocabulary Scale (BPVS) for single word comprehension; the PCA cohort was given the short-form RMT for words and faces, and the Concrete Synonyms test for single word comprehension. To enable comparisons between groups, we derived z-scores using age-appropriate mean and standard deviations from published normative data. Normative data for the BPVS are only available up to age 18, so we used data from a reference cohort of healthy older control participants who had also completed this task (mean age 65.3; SD 8.0) to generate z-scores for the tAD group. Mean (standard deviation) data are presented throughout the table unless otherwise indicated; maximum scores for neuropsychological tests are indicated in parentheses. Bold indicates significantly lower than healthy controls, $p<0.001$ unless otherwise specified; italics indicates significantly lower than tAD group, $p < 0.01$ unless otherwise specified (statistical data including 95% confidence intervals are presented in full in Table S1 in Supplementary Material online. Group comparisons for general and neuropsychological data are based on Fisher's exact test for categorical variables and analysis of variance (ANOVA) models for continuous variables: the far-right column gives the p-value for the main effect of diagnosis, and where this was significant, post-hoc between group comparisons are indicated in the relevant column. Group comparisons for ASA measures (see text and Figure 1) are based on non-parametric, bias corrected and accelerated 95% confidence intervals with 50000 bootstrap resamples, and p-values were also generated using a permutation approach that relaxes the assumptions of homoscedasticity and normality made by ANCOVA. Analyses for the main auditory scene analysis (ASA) tasks incorporated scores on the associated control tasks and peripheral hearing scores as covariates; analyses for the control tasks incorporated peripheral hearing score as a covariate. *Cerebrospinal fluid profiles of tau and beta-amyloid were available for thirteen patients with PCA and were consistent with Alzheimer's pathology in twelve cases, based on local reference ranges (total tau/beta-amyloid1-42 ratio > 1). **One participant with PCA showed clear response bias on the ASA-grouping task - this participant was removed from analysis of the ASA-grouping test. [1]data were not available for one participant with tAD; [2]data were not available for one participant with PCA; [3]data were not available for four healthy control participants. Administration of the graded naming test differed across groups: control and tAD participants were presented with items visually; participants with PCA were asked to name from verbal description. ASA, auditory scene analysis; Controls, healthy control group; F, female; M, male; MMSE, Mini-Mental State Examination score; N, number; PCA, patient group with posterior cortical atrophy; RMT, Recognition Memory Test; tAD, patient group with typical Alzheimer's disease: WASI, Wechsler Abbreviated Scale of Intelligence.

Table S4. Response accuracy across sequence types in the main auditory scene analysis tests

	**Controls**	**PCA**	**tAD**
**ASA-grouping task**			
Even	0.95 (0.06)	0.53 (0.30)	0.78 (0.25)
Uneven	0.91 (0.12)	0.59 (0.23)	0.78 (0.24)
**ASA-segregation task**			
Continuous	0.99 (0.03)	0.65 (0.29)	0.85 (0.24)
Intermittent	0.92 (0.13)	0.57 (0.25)	0.70 (0.31)

Mean (standard deviation) values are shown. An accuracy (hit rate) of 1.0 would signify perfect performance. In the control group, there was no difference between hit rates for even vs uneven sequences, $t(14) = 1.20$, $p = 0.25$). The difference in hit rats for continuous vs intermittent sequences was significant ($t(14) = 2.32$, $p = 0.036$), with this likely driven by the majority of controls achieving a perfect hit rate for the continuous stimuli. There was no difference in hit rates for even vs uneven sequences in the PCA group, $t(19) = -0.72$, $p = 0.478$, nor in hit rates for continuous vs intermittent sequences, $t(20) = 0.81$, $p = 0.43$. In the tAD group, there was no difference between hit rates for even vs uneven sequences, $t(20) = - 0.00$, $p = 1.00$; and no difference between hit rates for continuous vs intermittent sequences, $t(19) = 1.94$, $p = 0.068$. Controls, healthy control group; PCA, patient group with posterior cortical atrophy; tAD, patient group with typical Alzheimer’s disease.

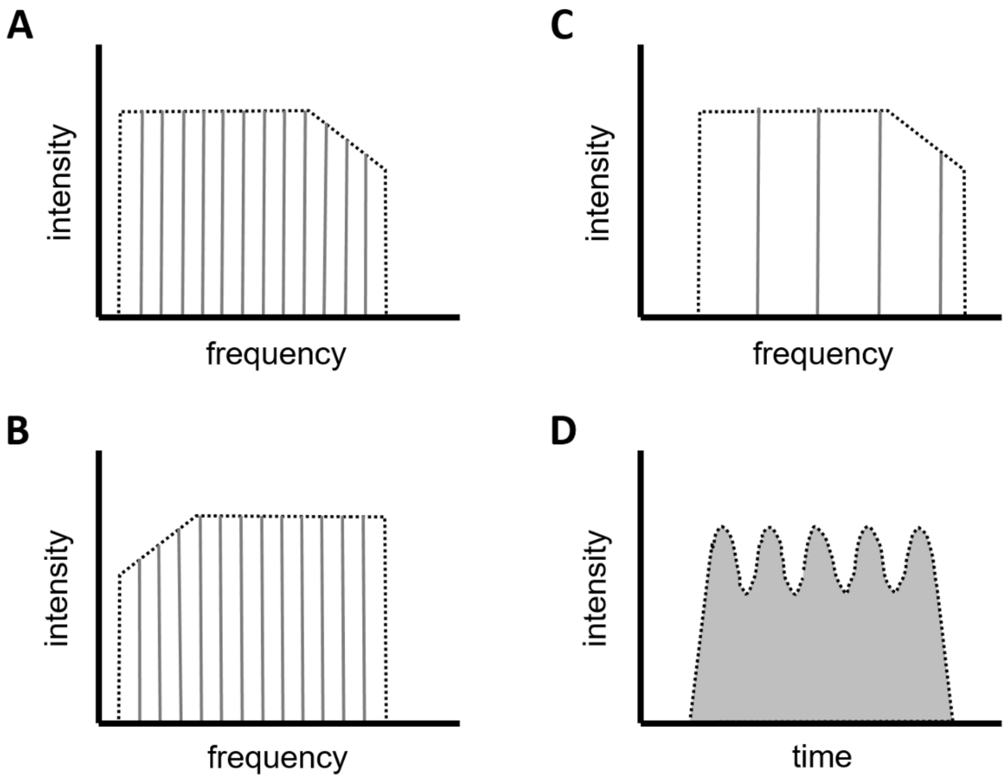

Figure S1. Diagram of acoustic structure of individual synthetic sound elements composing the stimulus sound sequences (see also Figure 1). Sound elements were based on harmonic series, schematised in the frequency domain in **A**, **B** and **C**; each series had a 'spectral shape' created by changing the envelope (shown as a dotted line) formed by the relative intensities of harmonics in the series. Within trials for the ASA-segregation test, sound elements had different spectral shapes (compare **A** and **B**) corresponding to the target and distractor timbres; while within trials in the ASA-grouping test, sound elements had different fundamental frequencies (compare **A** and **C**) corresponding to the target and distractor pitches. In the time domain (**D**), all sound elements across the experimental tests were ramped on and off and amplitude modulated using the same temporal parameters. Note that diagrams here are purely illustrative and not to scale.

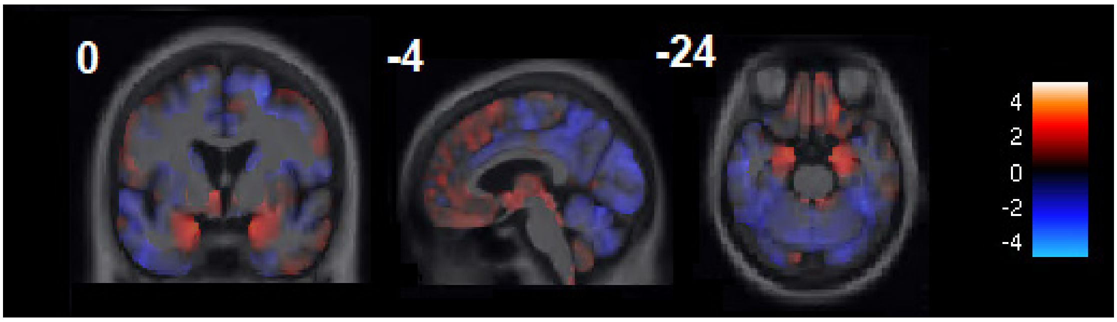

Figure S2. T-contrast effect maps showing associations between diagnosis and grey matter volume, adjusting for total intracranial volume, age, and sex. Warmer colours indicate greater atrophy in the patient group with typical Alzheimer's disease; cooler colours indicate greater atrophy in the group with posterior cortical atrophy. Maps are thresholded at $p < 0.001$ uncorrected over the whole brain and displayed on representative sections of a mean of all patients' T1-weighted brain magnetic resonance images. The plane of each section is indicated using Montreal Neurological Institute coordinates. The left cerebral hemisphere is displayed on the left in coronal and axial sections. These effect maps are unthresholded (i.e. not corrected for multiple comparisons) and so caution should be exercised when interpreting them, especially with respect to the main behavioural findings reported in the main manuscript: the maps are included here to corroborate that the two patient groups displayed the anticipated profiles of disease-related atrophy (i.e. predominantly hippocampal and mesial temporal volume loss in typical Alzheimer's disease; predominantly posterior cortical volume loss in posterior cortical atrophy).
